# Gender Differences in Perceived Family Involvement and Perceived Family Control during Emerging Adulthood: A Cross-Country Comparison in Southern Europe

**DOI:** 10.1007/s10826-021-02122-y

**Published:** 2021-10-23

**Authors:** M. C. García-Mendoza, A. Parra, I. Sánchez-Queija, J. E. Oliveira, S. Coimbra

**Affiliations:** 1 Department of Developmental and Educational Psychology, University of Seville, Seville, Spain; 2 Department of Geography and Spatial Planning. Faculty of Humanities, Education and Social Sciences, University of Luxembourg, Belval, Luxembourg; 3 Department of Psychology, Faculdade de Psicologia e de Ciências da Educação da Universidade do Porto, Porto, Portugal

**Keywords:** Perceived parental involvement, Perceived parental control, Emerging adulthood, Gender differences, Cross-national comparison

## Abstract

The aim of the present study was to explore gender differences in perceived parental involvement and perceived psychological and behavioral control during emerging adulthood in two Southern European countries (Spain and Portugal). Data were collected from 491 Portuguese and 552 Spanish undergraduate emerging adults (53.7 % women and 46.3 % men) aged between 18 and 29 years (*M* = 20.24 and SD = 2.12). Results indicated that women perceived higher levels of parental involvement than men in both countries, and men perceived more behavioral control than women in Portugal. Furthermore, gender was found to moderate the association between perceived parental involvement and perceived psychological and behavioral control differently in each country. Taken together, our findings suggest that gender-differentiated socialization patterns persist during emerging adulthood and that these patterns may be affected by the sociocultural context.

## Highlights

- Older children’s gender is a key variable that influences family relationships during emerging adulthood in southern Europe, being Spain and Portugal the focus of the present study.
- Women perceive higher levels of parental involvement than men in both countries, while men perceive higher levels of behavioral control than women exclusively in Portugal. No gender differences were found among either Portuguese or Spanish emerging adults in terms of perceived psychological control.
- Spanish emerging adults perceive higher levels of parental involvement and behavioral control than their Portuguese counterparts.
- Greater perceived parental involvement is associated with less perceived psychological control among both women and men, in both countries, although this association is stronger among women.
- Greater perceived parental involvement is associated with less perceived behavioral control in Spain (for both genders) and for women in Portugal.

## Transition to Adulthood and Family Roles in Southern Europe

The acquisition of adult roles is being increasingly delayed in industrialized countries as a result of the political, economic and social changes that have occurred in recent decades. These changes can be grouped into four major revolutions: the Technology Revolution, the Sexual Revolution, the Women’s Movement and the Youth Movement (Arnett, 2004). These phenomena prompted Arnett (2004, 2014) to propose the emergence of a new developmental stage between 18 and 29 years of age, called *emerging adulthood*. Emerging adults are legally adults, but have not yet acquired the responsibilities of adulthood. Despite the assumed generalization of this new life stage across and within societies, variations are likely to be observed in accordance with sociocultural features (Arnett, 2016a, 2016b, 2016c).

Since the transition to adulthood in Southern Europe occurs within the family context (Crocetti & Meeus, 2014), understanding how parent-child relationships evolve and eventually change during this life phase has become particularly relevant. In this respect, countries in this region are characterized by their “family welfare regimes”, in which one can observe low levels of youth employment, low social expenditure and strong traditional family ties (Vogel, 2002). The process of transition to adulthood is seen as a family project and is (almost) exclusively supported (economically, socially and emotionally) by the family (Albertini, 2010; Andrade, 2010). Considering the difficulties, the delays and the setbacks usually observed until the youth can establish him/herself in the labor market and make a living independently (Moreno & Marí-Klose, 2013), this is in fact, a period in which emerging adults may be most in need of family support (Hood et al., 2013). Accordingly, in comparison with their counterparts from other European regions, Southern European youths tend to attach greater importance to the role of the family during the coming of age process (Iacouvou, 2010; Moreno et al., 2012; Oliveira et al., 2014; Parra et al., 2019). The salient role played by the family may also be observed in the fact that these youths tend to spend this period of their lives under the parental roof, thereby extending the cohabitation period of the two generations (Coimbra & Mendonça, 2013; Oliveira et al., 2014; Mendonça et al., 2009; Parra et al., 2019). In Southern Europe, residential independence tends to be linked to prior acquisition of stability in the fields of love and employment (stable romantic and work relationships) (Buhl, 2007). However, attaining job stability has become particularly difficult for these youths (Organisation for Economic Co-operation and Development [OECD], 2018), many of whom do not acquire financial emancipation until their late 20 s (Eurostat, 2018). This delay in leaving the parental home and in the acquisition of adult roles and responsibilities results in an extension of the parent-child cohabitation period, which in turn affects family relationships and the overall family dynamics. As young people gradually acquire adult roles and responsibilities, a change in the way parents and their older children perceive each other also occurs (Mendonça & Fontaine, 2013a); their relationships gradually are transformed in search of a new balance, based on greater relational symmetry and equality between the two parties (Fosco et al., 2012; Manzi et al., 2012).

In this process, the beliefs, attitudes and behaviors of both parents and children concerning family relational dynamics are constructed and interpreted within their cultural and historical milieu (Harkness & Super, 2006). In this respect, Southern European countries such as Spain and Portugal present some socio-cultural and economic similarities. In both there is a prevalence of familistic values, preferences and attitudes towards leaving the family home, as well as an appreciation of the importance of family support during the coming of age period. Gender differences regarding family relationships also tend to present similar patterns in both countries. Emerging adult women tend to be more involved with their families and perceive a better family relationship (Marinho & Mena, 2012), described as warmer, closer, increasingly emotional and intimate (Parra et al., 2015). On their turn, emerging adult men tend to be less involved in family relationships and perceive family closeness or intimacy as intrusive (Marinho & Mena, 2012). In the economic sphere, some commonalities can also be observed since both countries recently underwent a severe economic crisis that had a negative impact on public youth policies and youth employment rates. Yet, despite such similarities regarding the relevant supportive role of the family during this life phase and the economic hurdles that both countries are facing, some cultural and socioeconomic nuances may also be observed in the way family relationships evolve and are interpreted during the coming of age period. Throughout history, geopolitical factors have created a stark separation between the two nations and maintained a salient set of economic and sociocultural differences (Gouveia & Ros, 2000; López, 2012; Roque, 1997; Telo & Torre, 2003; Torre & Jiméney, 2020). In the cultural domain, despite similar familistic values, in Portugal there is a greater tendency towards cooperation, consensus and the search for quality of life, whereas in Spain there is a stronger focus on competition, achievement and success (Hofstede, 2018). In the socioeconomic sphere, it is relevant to observe that at the beginning of 2019, youth unemployment was much higher in Spain than in Portugal (32.5% vs. 18.3%, respectively) (Eurostat, 2020). Such country specificities may translate into different timings of transitioning to the world of work and of gaining autonomy from the family of origin, which may affect the parental role in possibly distinct coming of age trajectories in the both countries.

## Parenting Dimensions During Emerging Adulthood

Studies focusing on the family context have mainly addressed two parental relationship dimensions: control and affect (Baumrind, 1971). Regarding control, it is necessary to clarify the conceptual difference between psychological control and behavioral control. Psychological control refers to parental behaviors that are intrusive and manipulative of the child’s feelings and thoughts, using methods such as guilt induction, whereas behavioral control refers to parental behaviors aimed at regulating the behavior of their children through strategies such as setting limits (Barber, 1996).

The study of parental control is particularly important during emerging adulthood since a key characteristic of this new stage involves the family bestowing a greater degree of autonomy on emerging adults (Padilla-Walker & Nelson, 2012). Some authors suggest that the levels of psychological and behavioral control imposed by parents during emerging adulthood are generally medium or low (Cui et al., 2019; Padilla-Walker et al., 2014; Reed et al., 2016; Soenens et al., 2009). Men also seem to perceive higher levels of psychological and behavioral control than women do, throughout their development and also specifically during this stage (Bean et al., 2006; García-Mendoza et al., 2018). Concerning the affective dimension, numerous studies have focused on warmth, affection and parental involvement. Some have found that young people perceive their family relationships to be characterized by high levels of affection (Gomez & McLaren, 2006), which even tends to increase when children start living outside the family home (García-Mendoza et al., 2017). Others stress the importance of continuing parental involvement during the transition to adulthood (Douglass, 2005), since this is associated with better adjustment among emerging adults (García-Mendoza et al., 2017). However, there is no consensus regarding the definition of what parental involvement actually is, and the term encompasses a wide variety of parental behaviors and practices (Henderson & Mapp, 2002). For the purposes of this study, it is defined as parental engagement expressed through parents spending time with their children and maintaining a high degree of concern about their lives.

Given that parental control is less intense during emerging adulthood, the role of parental involvement may be particularly important during this stage, since it is the variable that may traditionally fulfill the role of tracking and surveillance (Kerr & Stattin, 2000), a previously achieved through direct control. In fact, studies in this field suggest that young people generally perceive high levels of parental involvement during this life phase (Duchesne et al., 2007). Furthermore, some studies suggest that emerging adults associate higher-quality relationships with parents who have high levels of affection and autonomy promotion and who redefine the limits in a parent-child relationship decreasing levels of psychological control (Nelson et al., 2011). Studies carried out in Central and Southern Europe (León & Migliavacca, 2013; Moreno et al., 2012), also point that perceived parental involvement is significantly and negatively associated with perceived psychological and behavioral control.

With regard to gender, research focusing on family relationships during emerging adulthood suggests that, in general, parents’ behavior differs in accordance with their children’s gender (McKinney & Renk, 2008a, 2008b); parental involvement tends to be higher for women than for men (Ratelle et al., 2005). Indeed, emerging adult daughters tend to report higher levels of connectedness with their parents, maintaining a more emotionally and instrumentally interdependent relationship (Mendonça & Fontaine, 2013b; Sneed et al., 2006). Contrariwise, previous research observed that men tend to perceive higher levels of control than women (Bean et al., 2006; García-Mendoza et al., 2018). While the negative association between psychological control and affection within the family context has been amply reported (e.g., Romm et al., 2018), little is known about the role played by emerging adults’ gender as a possible moderator in this association. Although previous studies have clearly shown that children’s gender is a factor that has a decisive influence on their parents’ behavior (Cross & Madson, 1997; Kenny & Donaldson, 1991), to the best of our knowledge, no studies have yet taken into account the role played by gender in the relationship between these variables.

## The Present Study – Goals and Hypotheses

The general aim of this study is to expand current research on family relations during emerging adulthood from a culturally sensitive and a gender perspectives, moving beyond the prevalent models based on studies carried out in Northern European countries and the United States (Arnett, 2016b, 2016c). The specific aim is to analyze the relationship between perceived parental involvement, perceived psychological control and behavioral control from a gender perspective, and focusing on the cultural context of Southern Europe (specifically Spain and Portugal), where research is much scarcer.

In accordance with the previous studies mentioned above, the following hypotheses are formulated:

- In general, women are expected to perceive higher levels of parental involvement than men.
- In contrast, men are expected to perceive higher levels of control than women.
- In exploratory terms, we also hypothesize a possible gender difference in the relationship between parental control and parental involvement.
- Perceived parental involvement is expected to be significantly and negatively associated with perceived psychological and behavioral control, for both genders.
- Cross-cultural differences are expected regarding the former hypotheses and will be explored.

## Method

### Participants

A total of 1044 emerging adults from Spain and Portugal participated in the present study. The sample from Portugal comprised 491 Portuguese emerging adults (213 women and 278 men) aged between 18 and 30 years (*M* = 20.29; SD = 2.13) who formed part of the project Relações familiares em Portugal e ajustamento psicológico: investigação intercultural entre Espanha e Portugal. The sample from Spain comprised 552 Spanish emerging adults (282 women and 270 men) aged between 18 and 29 years (*M* = 20.20; SD = 2.10) who formed part of the La transición a la adultez en España: Estudio sobre las claves del ajuste psicosocial y fundamentos para su intervención preventiva (EDU2013-45687-R) and Estudio longitudinal secuencial sobre la transición a la adultez en España (RTI2018-097405-B-I00) projects. The majority (65.5%) of participants reported a medium level of family income, 21.4% reported a low family income and 13.1% reported a medium-high family income. Participants were university students recruited from two faculties (one in Spain and the other in Portugal) and were representative of the five major knowledge areas: Arts and Humanities, Sciences, Health Sciences, Social and Legal Sciences, and Engineering and Architecture.

### Data Collection Procedure

Firstly, faculty members from the two participating universities were contacted in order to explain the aims of the study and request permission to gather information from their students. Once students had agreed to take part in the study, they signed an informed consent form and anonymously, voluntarily and collectively completed a booklet containing the study instruments (~30 min. The study was approved by the Coordinating Committee for the Ethics of Biomedical Research in Andalusia (Spain) and by the Ethics Committee of Faculdade de Psicologia e de Ciencias da Educação da Universidade do Porto in Portugal (Refª 2017/10-2).

### Measures

#### Demographic variables

All participants indicated their age, gender and field of study. Perceived family income was measured using an ad hoc scale developed by the research team. Emerging adults were asked to rate their family’s income level on a 3-point scale ranging from 1 (perception of serious economic difficulties) to 3 (perception of having a high enough income to live comfortably).

#### Perceived family involvement

*Parental involvement* was measured using the parental involvement subscale of the Perceptions of Parents Scales (POPS), College Student Version (Grolnick et al., 1991; Robbins, 1994) translated into Spanish and Portuguese. Emerging adults rated six items on a 7-point Likert-type scale ranging from 1 (*not at all true*) to 7 (*very true*). Sample item: “My parent finds time to talk with me”. The Cronbach’s alpha for this scale was *α* = 0.85 in Portugal and *α* = 0.82 in Spain.

#### Perceived family control

*Psychological control* was measured using the corresponding subscale of the Parenting Styles Scale (Oliva et al., 2007) translated into Portuguese. Emerging adults rated eight items on a 6-point Likert-type scale ranging from 1 (*completely disagree*) to 6 (*completely agree*). Sample item: “My father/mother is always telling me what to do”. The Cronbach’s alpha for this scale was *α* = 0.87 in Portugal and *α* = 0.87 in Spain.

*Behavioral control* was measured using items from Kerr and Stattin’s Control Subscale (Kerr & Stattin, 2000) adapted for emerging adults and translated into Spanish and Portuguese. Emerging adults rated five items on a 6-point Likert-type scale ranging from 1 (*completely disagree*) to 6 (*completely agree*). Sample item: “My father/mother tries to set rules about what I do in my spare time”. The Cronbach’s alpha for this scale was *α* = 0.79 in Portugal and *α* = 0.77 in Spain.

### Data Analysis Procedure

The Statistical Package for the Social Sciences (SPSS), 24.0 was used for the data analysis. The first step in our analysis was to calculate the descriptive statistics and intercorrelations. As all variables correlated with each other, the second step was to perform a Multivariate Analysis of Variance (MANOVA). Whenever the MANOVA yielded a significant effect, subsequent one-way ANOVAs were performed. The third step was to analyze the possible moderating role of gender in the association between perceived parental involvement and control; thus, further hierarchical multiple regression analyses were performed for both countries separately, jointly adding gender and parental involvement, followed by their interaction term, as predictors of perceived psychological and behavioral control. All variables were centered for the analyses. The final step explored the significant interactions by using the procedure recommended by Aiken and West (1991). The Jose program (2013) was used to graphically represent the interactions. The graph obtained using this software divides the IV (perceived parental involvement) into three levels: high, medium, and low, using the mean as the medium value, one standard deviation above the mean as the high mean, and one standard deviation below the mean as the low mean (following Aiken & West, 1991) and draws a line for each of the dichotomous variables (men and women).

## Results

### Preliminary Analyses

The descriptive statistics and intercorrelations pertaining to the study variables are presented in Tables 1 and 2. An analysis of the correlations between the variables revealed that perceived parental involvement was negatively and significantly associated with both perceived behavioral and psychological control among women in Spain and Portugal. However, among men, perceived parental involvement correlated negatively and significantly with only perceived psychological control in both countries. Moreover, correlations above the diagonal (women) were stronger than those below the diagonal (men), suggesting gender moderation effects between perceived parental involvement and both perceived behavioral and psychological control.

**Table 1:** Descriptive statistics of study variables in Spain and Portugal

			Spain							Portugal				
	*M (SD)*		*M (SD)*	Min.	Max.	*S*	*K*	*M (SD)*		*M (SD)*	Min.	Max.	*S*	*K*
	Women	Men	Total					Women	Men	Total				
1. Perceived parental involvement	5.63 (1.17)	5.40 (1.16)	5.52 (1.17)	1	7	−1.09	1.11	5.51 (1.05)	5.14 (1.03)	5.34 (1.05)	2.33	7	−0.43	−0.60
2. Perceived behavioral control	2.26 (1.15)	2.33 (1.04)	2.29 (1.10)	1	6	0.89	0.34	1.96 (0.97)	2.17 (1.01)	2.05 (0.99)	0.80	5.20	0.94	0.10
3. Perceived psychological control	2.18 (1.17)	2.11 (0.99)	2.15 (1.08)	1	6	1.12	0.75	2.02 (1.06)	2.16 (0.95)	2.08 (1.01)	1	5.50	0.95	0.13

*M* Mean*, SD* Standard Deviation*,* *Min.* Minimum, *Max.* Maximum, *S* Skewness, *K* Kurtosis

**Table 2:** Intercorrelations among study variables by gender

	Spain			Portugal		
	1	2	3	1	2	3
1. Perceived parental involvement	–	−0.21**	−0.58**	–	−0.18*	−0.49**
2. Perceived behavioral control	−0.06	–	0.58**	0.10	–	0.64**
3. Perceived psychological control	−0.32**	0.53**	–	−0.27**	0.56**	–

Intercorrelations for women are shown above the diagonal; for men, below

*p < 0.01, **p < 0.001

### Differences According to Gender and Country for Perceived Parental Involvement and Perceived Psychological and Behavioral Control

The MANOVA indicated a significant effect of gender [Pillai’s Trace = 0.03; *F*(3,1040) = 10.34, *p* = 0.000, η^2^p = 0.03, Power = 0.999] and country [Pillai’s Trace = 0.021; *F*(3,1040) = 7.40, *p* = 0.000, η^2^*p* = 0.02, Power = 0.986], both with a small effect size. In contrast, no interaction effect between gender and country was observed [Pillai’s Trace = 0.00; *F*(3,1040) = 0.91, *p* = 0.435, η^2^p = 0.003, Power = 0.251]. When testing for the assumption of homogeneity of variance, Levene’s test confirmed the assumption for perceived parental involvement [*F*(3,1040) = 0.591; *p* = 0.591] and perceived psychological control [*F*(3,1040) = 2.409; *p* = 0.066], but indicated a violation of homogeneity of variance for perceived behavioral control.

The ANOVA suggested that gender is related to perceived parental involvement [*F*(1,1040) = 17.67, *p* < 0.001, η^2^p = 0.02, Power = 0.987], but with a small effect size. For perceived behavioral control, due to the violation of the homogeneity of variance assumption, Welch’s test was conducted, finding also a relationship with gender with a small effect size [*Welch’s F*(1,1029) = 5.26, *p* = 0.022, η^2^p = 0.00]. However, gender was found to exert no effect on perceived psychological control [*F*(1,1040) = 0.18, *p* = 0.670, η^2^p = 0.000, Power = 0.71]. Women reported slightly higher values in perceived parental involvement than men, while men reported slightly higher values in perceived behavioral control than women.

The ANOVA indicated that, for its part, country also is related to perceived parental involvement [*F*(1,1040) = 8.091, *p* = 0.005, η^2^p = 0.008, Power = 0.811] and perceived behavioral control [*Welch’s F*(1,1042) = 13.475, *p* < 0.001, η^2^p = 0.013], both with a small effect size, yet had no effect on perceived psychological control [*F*(1,1040) = 0.78, *p* = 0.379, η^2^p = 0.00, Power = 0.142]. Spanish emerging adults reported slightly higher values in perceived parental involvement and perceived behavioral control than their Portuguese counterparts (see Table 1).

### Gender as a Moderator of the Influence of Perceived Parental Involvement on Perceived Psychological and Behavioral Control

For Spain, the results of the hierarchical multiple regression analysis indicated that when perceived parental involvement and gender were included together, both variables added significantly to the prediction of perceived psychological control (*β* = −0.08, *p* = 0.034 for gender and *β* = −0.29, *p* < 0.001 for perceived parental involvement). The results of the regression equation also revealed a significant interaction effect [*F*(3,548) = 58.40, *p* < 0.001, *R*^*2*^ = 0.24]. Regarding perceived behavioral control, the results of the hierarchical multiple regression analysis revealed that when perceived parental involvement and gender were included together, only perceived parental involvement added significantly to the prediction of this variable (*β* = −0.14, *p* = 0.001). In this case, the results of the regression equation revealed a residual interaction effect [*F*(3,548) = 5.17*, p* = 0.06, *R*^*2*^ = 0.03].

For Portugal, the results revealed that only perceived parental involvement predicted perceived psychological control in the additive model (*β* = −0.26, *p* ≤ 0.001). Again, the results of the regression equation revealed a significant interaction effect [*F*(3,488) = 36.38, *p* = 0.003, *R*^*2*^ = 0.18]. Concerning perceived behavioral control, when perceived parental involvement and gender were included together, only gender was found to be a significant predictor (*β* = −0.10, *p* = 0.025). The results of the regression equation once again revealed a significant interaction effect [*F*(3,488) = 5.41, *p* = 0.003, *R*^*2*^ = 0.03] (see Table 3).

**Table 3:** Hierarchical regression analyses examining perceived parental involvement and gender as predictors of perceived psychological and behavioral control in Spain and Portugal

	Spain						Portugal					
	Perceived psychological control			Perceived behavioral control			Perceived psychological control			Perceived behavioral control		
Variable	B	SEB	*β*	B	SEB	*β*	B	SEB	*β*	B	SEB	*β*
MODEL 1: Additive												
Gender	0.17	0.08	0.08*	−0.04	0.09	−0.02	0.01	0.08	0.00	−0.19	0.09	−0.09*
Perceived parental involvement	−0.43	0.03	−0.46***	−0.13	0.04	−0.14***	−0.39	0.04	−0.41***	−0.05	0.04	−0.06
MODEL 2: Interactive												
Gender	0.17	0.08	0.08*	−0.04	0.09	−0.02	−0.00	0.08	−0.00	−0.20	0.09	−0.10*
Perceived parental involvement	−0.27	0.05	−0.29***	−0.06	0.06	−0.06	−0.25	0.06	−0.26***	0.09	0.06	0.10
Perceived parental involvement x Gender	−0.31	0.07	−0.24***	−0.15	0.08	−0.11	−0.24	0.08	−0.19**	−0.26	0.09	−0.21**
*R*^2^ Model 1	0.24			0.03			0.18			0.03		
*R*^2^ change (*p*)	0.03 (0.000)			0.01 (0.002)			0.1 (0.000)			0.02 (0.001)		

The gender variable was coded as women = 1 and men = 0

*p < 0.05, **p < 0.01, ***p < 0.001

**Fig. 1:**
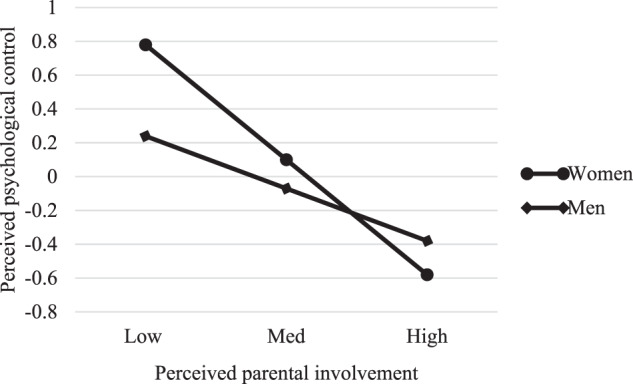
Moderating role of gender in the association between perceived parental involvement and perceived psychological control in Spain

**Fig. 2:**
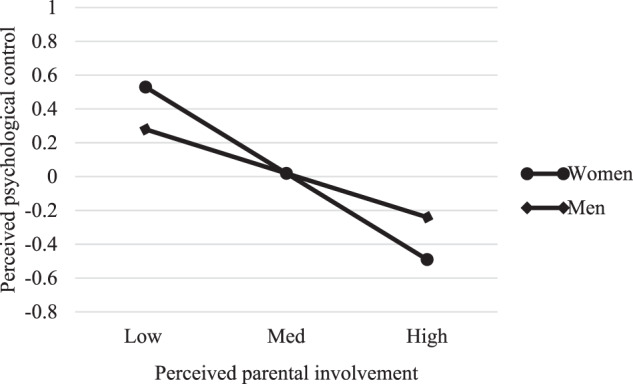
Moderating role of gender in the association between perceived parental involvement and perceived psychological control in Portugal

**Fig. 3:**
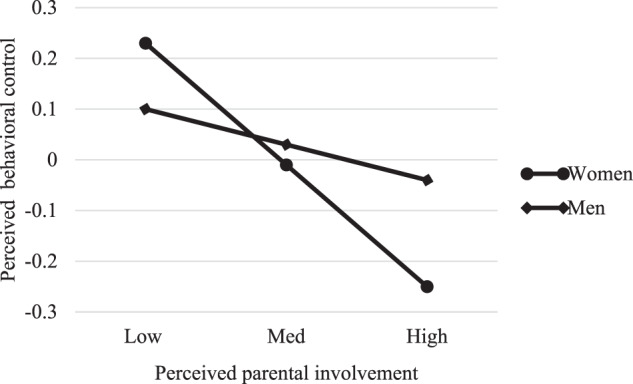
Moderating role of gender in the association between perceived parental involvement and perceived behavioral control in Spain

**Fig. 4:**
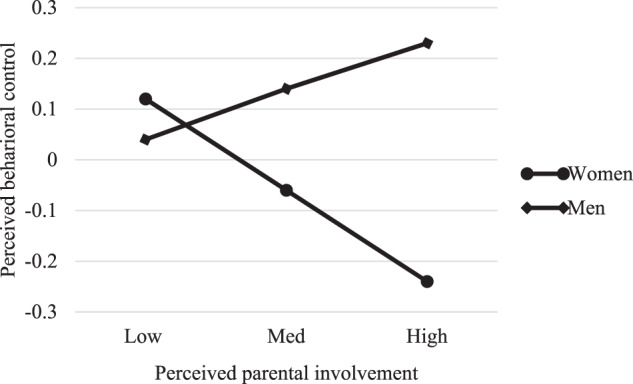
Moderating role of gender in the association between perceived parental involvement and perceived behavioral control in Portugal

We graphed the results to interpret the interactions. Spain and Portugal followed a similar pattern in relation to perceived psychological control: higher perceived parental involvement was associated with lower perceived psychological control for both women and men, although the slope for women was sharper, suggesting that this relationship is stronger among women (see Figs. 1 and 2). Nevertheless, while the pattern for perceived psychological control was replicated for perceived behavioral control in Spain, in Portugal it was different: for Portuguese emerging adult women, high perceived parental involvement was associated with a low perception of behavioral control, whereas for Portuguese emerging adult men, high perceived parental involvement was associated with a high perception of behavioral control (see Figs. 3 and 4).

## Discussion

The relationship between parental affection and control during emerging adulthood has received very little attention to date, particularly from a gender perspective. Thus, one of the main goals of this study was to contribute to a fuller understanding of family relational dynamics during this specific period of time, from a gender perspective. The other main goal was to address this same issue from a specific cultural perspective, that of Southern Europe, which has been absent from most family relations models constructed over recent decades. By focusing on two Southern European countries (Spain and Portugal), the present study was also able to address possible differences between the two cultures.

Regarding country-based differences in how young people perceive family relationships, our results suggest that Spanish emerging adults perceive higher levels of parental involvement and behavioral control than their Portuguese counterparts. This finding suggests that, even within a more global Southern European model, different patterns or sub-models may be found, possibly reflecting different constructions and interpretations of family relationships. It is true that Portugal and Spain share some common cultural features: they are both familistic and fairly traditional countries in terms of family relations. This usually means that caring for others, respecting norms, avoiding uncertainty and enjoying everyday life with restraint is more important than individual achievement, deep societal change, pragmatism or preparing for the future (Hofstede, 2018). However, some cultural differences also exist between the two countries that may have an impact on parent-child relationships during the coming of age process. In this respect, the more salient tendency in the Spanish culture towards achievement and success (in comparison with the Portuguese culture) perhaps prompts parents to be more involved and exert greater behavioral control over their older children, with the aim of directing their lives towards what parents consider the most appropriate pathway to success. Nevertheless, despite the cultural differences observed, there are mostly similarities between the two cultures.

Regarding gender, the data also indicate the existence of differences, albeit minor ones. Women perceived higher levels of parental involvement in both countries. In terms of perceived family control, differences were observed in perceived behavioral control only, which was higher in men, but only among Portuguese emerging adults. These results suggest that the differential socialization patterns for sons and daughters observed during childhood and adolescence continue to be prevalent during emerging adulthood. A large body of research has highlighted the differential socialization patterns that, from the initial years of development, prompt parents to emphasize the development of intimate relationships and emotions more with their daughters than with their sons (e.g., Brody & Hall, 2000; Eisenberg et al., 1998). Consequently, in this developmental stage, women have more contacts (Sneed et al., 2006), feel more united with their parents (Kenny & Donaldson, 1991) and perceive more social support from the family than men (Adamczyk, 2016; Duru, 2007).

Regarding the relationship between involvement and control, both Spanish and Portuguese emerging adults who perceived higher levels of parental involvement also perceived lower levels of psychological parental control. These results are consistent with those reported by previous studies, which found a similar negative association between psychological control and warmth among young people (Romm et al., 2018). These results seem to confirm that, during emerging adulthood, a new balance is established between parents and their children that results in more symmetrical and horizontal relationships (Fosco et al., 2012; Manzi et al., 2012). Parents maintain high involvement levels, but exercise less psychological control over their emerging adult children. Thus, while the strong family ties characteristic of Southern European countries is maintained, new boundaries are also being set in parent-child relationships (Giuliano, 2007; Nelson et al., 2011).

The results also reveal that the relationship between perceived parental involvement and perceived psychological control is moderated by gender in both countries, following a similar pattern. Greater perceived parental involvement is associated with less perceived psychological control among both women and men, although among women this association is stronger. One possible explanation for this finding lies in the relationship between the two variables. As mentioned earlier, women perceive more parental involvement than men and similar levels of psychological control in both countries. Therefore, it is perhaps not surprising that, for them, perceiving high levels of parental involvement results in a stronger relationship between the two variables. When parents spend time with their emerging adult children and show concern over their life issues, they tend to do it in a way that is increasingly symmetrical and removed from psychological control patterns. Thus, the fact that parents become more involved with their daughters renders this reduction in control more drastic.

In relation to perceived behavioral control, graphs showed that, in Spain, the trend followed by the association between this variable and perceived parental involvement was similar to the one for perceived psychological control. But why is this association different in Portugal? Fig. 4 indicates that, among Portuguese emerging adult men, high perceived parental involvement is associated with a high perception of behavioral control exercised by parents. This perception of high behavioral control is perhaps due to the fact that Portuguese men are even more independent or have more desire for autonomy than their Spanish counterparts and perceive high parental involvement as control. Moreover, while parental warmth is a dimension of parenting that is positively valued universally, parental control may vary more across cultures with respect to its potential consequences (Deater-Deckard et al., 2011). In any case, these results should be read with caution, as correlations between variables showed that relationships between perceived behavioral control and perceived parental involvement were not significant for men.

The conceptual distinction between behavioral and psychological control has been recognized across different nationalities and ethnicities (Wang et al., 2007) and its impact may be expected to vary from culture to culture (Chou & Chou, 2018). The differences observed in gender moderating patterns further highlight the importance of cultural contexts, underscoring, in our case, the effect of cultural specificities (Spanish and Portuguese) on family relationships.

Some limitations of the study should be acknowledged. Firstly, a cross-sectional design was used, meaning that no causal inferences can be made. In future research it would be interesting to use a longitudinal design that would allow us to analyze the development of parent-child relationships throughout emerging adulthood, in order to observe, for instance, the evolution of the relationship between perceived parental involvement and perceived family control, and the moderating effect of gender in this relationship during this period. Secondly, the entire sample was made up of university students, meaning that the results cannot be generalized to the non-student population. Despite a significant increase in the number of young university students over recent decades, many young people still choose not to go or do not have the means to go to university (Arnett, 2016a; Brock, 2010; Lee & Goldstein, 2016). Furthermore, all measures were self-reported, which can lead to an overestimation of some of the associations between the variables studied, and we have not considered variables such us level of family income, that could influence the results. Finally, our data came from a single source, emerging adults, and did not take parents’ views into account. It would be interesting for future research to gather data from different informants (e.g., children and parents), with the results being analyzed simultaneously to provide a broader, dyadic and more complex view of parent-child relationships during emerging adulthood.

Despite these limitations, this study makes a relevant practical contribution to the studies of coming of age by highlighting the importance of parental involvement even during emerging adulthood. From previous research, we know that control (psychological and behavioral) has negative consequences for the development of older children (Costa et al., 2015; García-Mendoza et al., 2018; Nelson et al., 2011) and our data indicate that perceived greater parental involvement is related to less control, especially in the case of females. This result is particularly interesting since it underlines the relevance of parental involvement as a key element to be addressed in psychosocial interventions with families with children in this developmental stage. This may be even more important, when the social and financial impact of COVID 19 pandemic will most probably narrow even more the opportunities of emerging adults to find a job or become independent. Last but not least, a most needed contribution is made to further our knowledge of the Southern European model of transitioning to adulthood, taking into consideration cross-cultural similarities and differences between the countries under analysis (Spain and Portugal) as well as culturally constructed gender differences. In fact, this article presents one of the first cross-national studies focused on Southern European countries, thus making significant ecologically-embedded contributions to the understanding of family relationships during emerging adulthood. It provides evidence that the gender differential socialization patterns documented in previous stages of development (e.g., Brody & Hall, 2000; Eisenberg et al., 1998) apparently persist during emerging adulthood. Furthermore, these patterns tend to differ in some respects depending on the cultural milieu, even between fairly similar contexts. Thus, if we are truly to understand young people’s transition towards adulthood, then we must take the sociocultural context in which the transition occurs into account (Arnett, 2010, 2016c). Expanding these studies to other cultures would also help us draw firmer conclusions about family relations during the coming of age process. Finally, this study emphasizes the importance of not assuming that parenting behavior has the same meaning across cultures, and of continuing to pay close attention to the challenges of measurement and interpretation (Deater-Deckard et al., 2011).

## References

[CR1] Aiken, L. S., & West, S. G. (1991). Multiple Regression: Testing and Interpreting Interactions. Sage. 10.1037/10520-147.

[CR2] Adamczyk K (2016). An investigation of loneliness and perceived social support among single and partnered young adults. Current Psychology.

[CR3] Albertini M (2010). La ayuda de los padres españoles a los jóvenes adultos. El familismo español en perspectiva comparada. [The help provided by Spanish parents to young adults. A comparative approach to Spanish familialism]. Revista de Estudios de Juventud.

[CR4] Andrade, C. (2010). Trabalho e família na transição para a idade adulta. [Work and Family in the Transition to Adulthood]. LivPsic.

[CR5] Arnett, J. J. (2004). Emerging adulthood: the winding road from the late teens through the twenties. Oxford University Press. 10.1093/acprof:oso/9780199929382.001.0001.

[CR6] Arnett JJ (2010). Oh, grow up! Generational grumbling and the new life stage of emerging adulthood. Commentary on Trzesniewski & Donnellan. Perspectives on Psychological Science.

[CR7] Arnett JJ (2014). Presidential address: The emergence of emerging adulthood: A personal history. Emerging Adulthood.

[CR8] Arnett JJ (2016). Does emerging adulthood theory apply across social classes? National data on a persistent question. Emerging Adulthood.

[CR9] Arnett JJ (2016). Life stage concepts across history and cultures: Proposal for a new field on indigenous life stages. Human Development.

[CR10] Arnett, J. J. (2016c). The neglected 95%: Why American psychology needs to become less American. In A. E. Kazdin (Ed.), Methodological issues and strategies in clinical research, (4th ed., pp. 115–132). American Psychological Association. 10.1037/14805-008.

[CR11] Barber BK (1996). Parental psychological control: Revisiting a neglected construct. Child Development.

[CR12] Baumrind D (1971). Current patterns of parental authority. Developmental Psychology Monograph.

[CR13] Bean RA, Barber BK, Crane DR (2006). Parental support, behavioral control, and psychological control among African American youth. The relationships to academic grades, delinquency, and depression. Journal of Family Issues.

[CR14] Brock T (2010). Young adults and higher education: Barriers and breakthroughs to success. The Future of Children.

[CR15] Brody, L., & Hall, J. (2000). Gender, emotion, and expression. In M. Lewis & J. Haviland (Eds.), Handbook of emotions (pp. 338–349). Guilford Press.

[CR16] Buhl HM (2007). Well-being and the child–parent relationship at the transition from university to work life. Journal of Adolescent Research.

[CR17] Coimbra S, Mendonça MG (2013). Intergenerational solidarity and satisfaction with life: Mediation effects with emerging adults. Paidéia.

[CR18] Costa S, Soenens B, Gugliandolo MC, Cuzzocrea F, Larcan R (2015). The mediating role of experiences of need satisfaction in associations between parental psychological control and internalizing problems: A study among Italian college students. Journal of Child and Family Studies.

[CR19] Chou CP, Chou EPT (2018). Perceived parental psychological control and behavioral control among emerging adults: A cross-cultural study between the US and Taiwan. Current Psychology.

[CR20] Crocetti E, Meeus W (2014). “Family comes first!” Relationships with family and friends in Italian emerging adults. Journal of Adolescence.

[CR21] Cross SE, Madson L (1997). Models of the self: self-construals and gender. Psychological Bulletin.

[CR22] Cui M, Darling CA, Coccia C, Fincham FD, May RW (2019). Indulgent parenting, helicopter parenting, and well-being of parents and emerging adults. Journal of Child and Family Studies.

[CR23] Deater-Deckard K, Lansford JE, Malone PS, Alampay LP, Sorbring E, Bacchini D, Bombi AS, Bornstein MH, Chang L, Di Giunta L, Dodge KA, Oburu P, Pastorelli C, Skinner AT, Tapanya S, Tirado LMU, Zelli A, Al-Hassan SM (2011). The association between parental warmth and control in thirteen cultural groups. Journal of Family Psychology.

[CR24] Douglass, C. B. (2005). “We’re fine at home”: Young people, family and low fertility in Spain. In C. B. Douglass (Ed.), Barren states: The population “implosion” in Europe, (1st ed., pp. 183–207). Routledge. 10.4324/9781003084761.

[CR25] Duchesne S, Ratelle CF, Larose S, Guay F (2007). Adjustment trajectories in college science programs: Perceptions of qualities of parents’ and college teachers’ relationships. Journal of Counseling Psychology.

[CR26] Duru E (2007). Re-examination of the psychometric characteristics of the multidimensional scale of perceived social support among Turkish university students. Social Behavior and Personality: An International Journal.

[CR27] Eisenberg N, Cumberland A, Spinrad TL (1998). Parental socialization of emotion. Psychological Inquiry.

[CR28] Eurostat (2018). Estimated average age of young people leaving the parental household by sex. Products Datasets. https://appsso.eurostat.ec.europa.eu/nui/show.do?dataset=yth_demo_030&lang=en.

[CR29] Eurostat (2020). Youth unemployment rate by sex. Products Datasets. https://ec.europa.eu/eurostat/databrowser/view/tesem140/default/table?lang=en.

[CR30] Fosco GM, Caruthers AS, Dishion TJ (2012). A six-year predictive test of adolescent family relationship quality and effortful control pathways to emerging adult social and emotional health. Journal of Family Psychology.

[CR31] García-Mendoza MC, Parra Á, Sánchez-Queija I (2017). Relaciones familiares y ajuste psicológico en adultos emergentes universitarios españoles. [Family relationships and psychological adjustment in Spanish undergraduated emerging adults]. Psicología Conductual/Behavioral Psychology. Journal.

[CR32] García-Mendoza, M. C., Sánchez-Queija, I., & Parra Jiménez, Á. (2018). The Role of Parents in Emerging Adults’ Psychological Well-Being: A Person-Oriented Approach. Family Process. Advanced online publication. 10.1111/famp.12388. 10.1111/famp.12388 30198562

[CR33] Giuliano P (2007). Living arrangements in Western Europe: Does cultural origin matter?. Journal of the European Economic Association.

[CR34] Gomez R, McLaren S (2006). The association of avoidance coping style, and perceived mother and father support with anxiety/depression among late adolescents: Applicability of resiliency models. Personality and Individual Differences.

[CR35] Gouveia VV, Ros M (2000). Hofstede and Schwartz s models for classifying individualism at the cultural level: their relation to macro-social and macro-economic variables. Psicothema.

[CR36] Grolnick WS, Ryan RM, Deci EL (1991). Inner resources for school achievement: Motivational mediators of children’s perceptions of their parents. Journal of Educational Psychology.

[CR37] Harkness, S., & Super, C. M. (2006). Themes and variations: Parental ethnotheories in Western cultures. In Rubin, K. (Ed.), Parenting beliefs, behaviors, and parent-child relations: A cross-cultural perspective (pp. 61–79). Psychology Press.

[CR38] Henderson A, Mapp KL (2002). A new wave of evidence: The impact of school, family, and community connections on student achievement. Annual Synthesis.

[CR39] Hofstede, G. (2018, March 24). Country comparison. Hofstede Insights. https://www.hofstede-insights.com/country-comparison/portugal,spain/.

[CR40] Hood K, Brevard J, Nguyen AB, Belgrave F (2013). Stress among African American emerging adults: The role of family and cultural factors. Journal of Child and Family Studies.

[CR41] Iacouvou M (2010). Leaving home: Independence, togetherness and income in Europe. Advances in Life Course Research.

[CR42] Jose, P. E. (2013). Doing Statistical Mediation and Moderation. Guilford Press.

[CR43] Kenny ME, Donaldson GA (1991). Contributions of parental attachment and family structure to the social and psychological functioning of first-year college students. Journal of Counseling Psychology.

[CR44] Kerr M, Stattin H (2000). What parents know, how they know it, and several forms of adolescent adjustment: further support for a reinterpretation of monitoring. Developmental Psychology.

[CR45] Lee CYS, Goldstein SE (2016). Loneliness, stress, and social support in young adulthood: Does the source of support matter?. Journal of Youth and Adolescence.

[CR46] León M, Migliavacca M (2013). Italy and Spain: still the case of familistic welfare models?. Population Review.

[CR47] López, V. (2012). De Espanha nem bom vento nem bom casamento. [From Spain neither good wind nor good marriage]. A Esfera dos Livros.

[CR48] Manzi C, Regalia C, Pelucchi S, Fincham FD (2012). Documenting different domains of promotion of autonomy in families. Journal of Adolescence.

[CR49] Marinho L, Mena P (2012). Separation-individuation of Portuguese emerging adults in relation to parents and to the romantic partner. Journal of Youth Studies.

[CR50] McKinney C, Renk K (2008). Differential parenting between mothers and fathers: Implications for late adolescents. Journal of Family Issues.

[CR51] McKinney C, Renk K (2008). Multivariate models of parent-late adolescent gender dyads: The importance of parenting processes in predicting adjustment. Child Psychiatry and Human Development.

[CR52] Mendonça M, Andrade C, Fontaine AM (2009). Transição para a idade adulta e adultez emergente: Adaptação do Questionário de Marcadores de Adultez junto de jovens Portugueses. [Transition to adulthood: Validation of the Questionnaires of Adulthood Markers in a Portuguese sample of emerging adults]. Psychologica.

[CR53] Mendonça M, Fontaine AM (2013). Filial maturity in young adult children: The validity of the filial maturity measure and the role of adult transitions. TPM: Testing, Psychometrics, Methodology in Applied Psychology.

[CR54] Mendonça M, Fontaine AM (2013). Late nest leaving in Portugal: Its effects on individuation and parent–child relationships. Emerging Adulthood.

[CR55] Moreno, A., López, A., & Segado, S. (2012). La transición de los jóvenes a la vida adulta: crisis económica y emancipación tardía. [The transition of young people to adult life: economic crisis and late emancipation]. Barcelona: Obra Social “ La Caixa”.

[CR56] Moreno L, Marí-Klose P (2013). Youth, family change and welfare arrangements: Is the South still so different?. European Societies.

[CR57] Nelson LJ, Padilla-Walker LM, Christensen KJ, Evans CA, Carroll JS (2011). Parenting in emerging adulthood: An examination of parenting clusters and correlates. Journal of Youth and Adolescence.

[CR58] Organisation for Economic Co-operation and Development (2018). Panorama de la Educación. Indicadores de la Organización para la Cooperación y el Desarrollo Económicos 2018. Informe Español. [Panorama of Education. Indicators of the Organization for Economic Cooperation and Development 2018. Spanish Report.]. Secretaría General Técnica. https://www.todofp.es/dam/jcr:a4f4282f-cadc-4bb9-9faf-d2a6f7da7973/panorama%20de%20la%20educacion%202018-final.pdf.

[CR59] Oliva A, Párra Á, Sánchez-Queija I, López F (2007). Estilos educativos materno y paterno: Evaluación y relación con el ajuste adolescente. [Maternal and paternal parenting styles: evaluation and relationships with adolescent adjustment]. Anales de Psicología.

[CR60] Oliveira JE, Mendonça M, Coimbra S, Fontaine AM (2014). Family support in the transition to adulthood in Portugal – Its effects on identity capital development, uncertainty management and psychological well-being. Journal of Adolescence.

[CR61] Padilla-Walker LM, Nelson LJ (2012). Black hawk down?: Establishing helicopter parenting as a distinct construct from other forms of parental control during emerging adulthood. Journal of Adolescence.

[CR62] Padilla-Walker LM, Nelson LJ, Knapp DJ (2014). “Because I’m still the parent, that’s why!” parental legitimate authority during emerging adulthood. Journal of Social and Personal Relationships.

[CR63] Parra A, Oliva A, Reina MC (2015). Family relationships from adolescence to emerging adulthood: A longitudinal study. Journal of Family Issues.

[CR64] Parra Á, Sánchez-Queija I, García-Mendoza MC, Coimbra S, Egídio Oliveira J, Díez M (2019). Perceived parenting styles and adjustment during emerging adulthood: A cross-national perspective. International Journal of Environmental Research and Public Health.

[CR65] Ratelle CF, Larose S, Guay F, Senécal C (2005). Perceptions of parental involvement and support as predictors of college students’ persistence in a science curriculum. Journal of Family Psychology.

[CR66] Reed K, Duncan JM, Lucier-Greer M, Fixelle C, Ferraro AJ (2016). Helicopter parenting and emerging adult self-efficacy: Implications for mental and physical health. Journal of Child and Family Studies.

[CR67] Robbins, R. J. (1994). An assessment of perceptions of parental autonomy support and control: Child and parent correlates. [Unpublished Doctoral Dissertation]. Department of Psychology, University of Rochester.

[CR68] Roque, M. A. (1997). Identidades y conflicto de valores: Diversidad y mutación social en el Mediterráneo. [Identities and values conflict: diversity and social mutation in the Mediterranean]. Icaria.

[CR69] Romm KF, Barry CM, Kotchick BA, DiDonato TE, Barnett JE (2018). Parental psychological control and identity: The roles of warmth, gender, and ethnicity. Journal of Adult Development.

[CR70] Sneed J, Johnson J, Cohen P, Gilligan C, Chen P, Crawford T, Kasen S (2006). Gender differences in the age-changing relationhip between instrumentality and family contact in emerging adulthood. Developmental Psychology.

[CR71] Soenens B, Vansteenkiste M, Sierens E (2009). How are parental psychological control and Autonomy‐Support related? A cluster‐analytic approach. Journal of Marriage and Family.

[CR72] Telo, A. & Torre, H. (2003). Portugal y España en los Sistemas Internacionales Contemporáneos. [Portugal and Spain in the comtemporany international systems]. Editora Regional de Extremadura.

[CR73] Torre, H. & Jiméney, C. (2020). Historia de una diferencia: Portugal y España - Ayer y hoy (1807–2019). [History of a difference: Portugal and Spain – Yerterday and today (1807-2019)]. Silex Ediciones.

[CR74] Vogel J (2002). European welfare regimes and the transition to adulthood: A comparative and longitudinal perspective. Social Indicators Research.

[CR75] Wang Q, Pomerantz EM, Chen H (2007). The role of parents’ control in early adolescents’ psychological functioning: A longitudinal investigation in the United States and China. Child Development.

